# Eddy Current Rail Inspection Using AC Bridge Techniques

**DOI:** 10.6028/jres.118.007

**Published:** 2013-02-26

**Authors:** Ze Liu, Andrew D Koffman, Bryan C Waltrip, Yicheng Wang

**Affiliations:** 1 Beijing Jiaotong University, Beijing, P.R. China, 100044; 2 National Institute of Standards and Technology, Gaithersburg, MD 20899

**Keywords:** AC bridges, demodulation, digital lock-in amplifier, Eddy current rail inspection

## Abstract

AC bridge techniques commonly used for precision impedance measurements have been adapted to develop an eddy current sensor for rail defect detection. By using two detection coils instead of just one as in a conventional sensor, we can balance out the large baseline signals corresponding to a normal rail. We have significantly enhanced the detection sensitivity of the eddy current method by detecting and demodulating the differential signal of the two coils induced by rail defects, using a digital lock-in amplifier algorithm. We have also explored compensating for the lift-off effect of the eddy current sensor due to vibrations by using the summing signal of the detection coils to measure the lift-off distance. The dominant component of the summing signal is a constant resulting from direct coupling from the excitation coil, which can be experimentally determined. The remainder of the summing signal, which decreases as the lift-off distance increases, is induced by the secondary eddy current. This dependence on the lift-off distance is used to calibrate the differential signal, allowing for a more accurate characterization of the defects. Simulated experiments on a sample rail have been performed using a computer controlled X-Y moving table with the X-axis mimicking the train’s motion and the Y-axis mimicking the train’s vibrational bumping. Experimental results demonstrate the effectiveness of the new detection method.

## 1. Introduction

Train derailment accidents still happen often despite all available modern technologies. According to the train accident statistic data from the U.S. Federal Railroad Administration Office of Safety, the number of U.S. train accidents related to track problems for 2009, 2010, and 2011 were 630, 624, and 637, respectively [1]. Among many known possible causes including rail problems, signaling, communication, mechanical and electrical failure, and train operation, the rail defect is one of the main reasons for railway accidents [2]. It is challenging to develop effective and efficient rail defect detection techniques, which are needed to improve the safety of the railway transportation systems, especially for railways where the traffic density, train speed, and loads are high. For some busy tracks, the traffic density may leave a time gap as small as 8 minutes between running trains. This time gap is too limited for the currently-available rail inspection equipment to perform effective testing [3]. Ultrasonic-based techniques are the most common approach used in the field for rail defect detection, conducted on either an inspection train or a special inspection handcart. A major limitation associated with ultrasonic inspection is that the ultrasonic transducer must make close contact with the rail in order for ultrasonic wave transmission to occur. This requirement limits the speed of the inspection train, and significantly reduces the track usage efficiency [4–7]. For handcart mounted ultrasonic inspection, it is not convenient to move the handcart on and off the track when the time lag between trains falls below 10 minutes. An ideal solution is a real-time on-train inspection technology that can be mounted directly on the passenger or freight trains.

Recent studies [8,9] indicate that eddy current testing, among known physical test techniques (ultrasonic, optical, electromagnetic, thermal, and radiation), is a promising technology for developing an on-train real-time rail inspection system. This method does not require direct rail contact, but it involves small signal detection in noisy environments. Rail tracks are part of a communication circuit used to locate the train positions on the track [10]. The tracks may also carry the return current of locomotive motors which can be as high as hundreds of amperes [11]. Liu *et al.* [12] experimented with a conventional eddy current sensor consisting of an excitation coil and a single detection coil for simulated rail defect detection. They discuss the difficulty in extracting the defect-modulated signal. The output of the detection coil is dominated by the direct induced voltage from the excitation coil, and the voltage variation caused by the defect-induced eddy current change is buried in the large baseline signal at the same frequency. Another difficulty is that the distance between the eddy current sensor and the rail constantly varies due to vibrations and bumping, causing a “lift-off” effect affecting characterization of the defects. Various methods have been invented to deal with the lift-off effect with limited success. H. M. Thomas *et al.* [13] developed a rail inspection eddy current system in which the sensors are placed on a sled carrier to guide the eddy current probes. This mechanical method can reduce the influence of lift-off variations from the test surface, but the detection signal can still be affected by the sled carrier’s lift-off and vibrations caused by hard rolling contact with the rail. G. Y. Tian *et al.* [14] investigated lift-off invariance observed within narrow time windows in pulsed eddy-current signals. W. Yin *et al.* [15] explored the lift-off independence of the phase spectra of a double air-cored sensor and a ferrite U-cored sensor based on theoretical and experimental investigations. These indirect methods, however, lack sensitivity to extract the defect-induced signals and have proven difficult to be adopted in field applications.

The new eddy current method investigated in this paper employs conventional AC bridge techniques [16] to balance out the large baseline signals. With an excitation coil and two detection coils configured as a three winding transformer, forming a classical four-arm bridge with two known impedances, the bridge differential error signal can be selectively amplified and can sensitively detect rail defects using a digital lock-in amplifier algorithm [17,18]. The lock-in amplifier can act as a very narrow band pass filter (BPF) array which can extract the defect signal based only on the specific excitation reference signal. By selecting the optimized excitation frequencies, the lock-in amplifier method can easily reject the noise from the track circuit and the locomotive motor return current. The lift-off effect of the bridge sensor due to vibrations can also be measured using the summing signal of the detection coils and be used to dynamically compensate the differential signal to more accurately reflect the defect characteristics.

## 2. Measurement Principle

Let us first consider a typical eddy current sensor comprising one excitation coil and one detection coil, placed above a rail with the coil axes perpendicular to the rail surface and with an AC current of frequency *ω* fed into the excitation coil. The primary magnetic field of the excitation coil induces an eddy current in the rail, which in turn produces a secondary magnetic field. These time-varying fields together induce a voltage in the detection coil. A near-surface defect in the rail head will perturb the local eddy current distribution, leading to a small change in the induced voltage of the detecting coil.

The eddy current distribution below the rail surface can be estimated as follows. For optimal detection, the excitation frequency is typically in the range of several hundred Hz to several MHz [12], and in this low frequency region, the density of free electric charge in the rail is negligible. The magnetic field intensity, H, at depth, *x*, below the surface along the excitation coil axis can then be calculated according to electromagnetic theory [19]:

(1) $$\frac{d^{2}H}{dx^{2}}=j\omega \mu \sigma H,$$

where *σ* is the conductivity, and *µ* is the relative magnetic permeability. The induced eddy current density is:

(2) $$J=J_{0}e^{-\frac{1+j}{\sqrt{2}}\sqrt{\omega \mu \sigma }x}=J_{0}e^{-\sqrt{\frac{\omega \mu \sigma }{2}}x}e^{-j\sqrt{\frac{\omega \mu \sigma }{2}}x}.$$

It can be seen from Eq. (2) that as the depth *x* increases, the eddy current strength decreases at a rate dependent on the excitation frequency. This shows the eddy current method is mostly sensitive to defects near the surface and it also suggests that by using different frequencies, defects at various depths can be detected.

We use two detection coils instead of just one as in a typical sensor. This allows us to balance out the large baseline signals corresponding to a normal rail. The differential signal of the two coils is then related to the defects.

## 3. Detection Bridge Design and Signal Processing

**Fig. 1:**
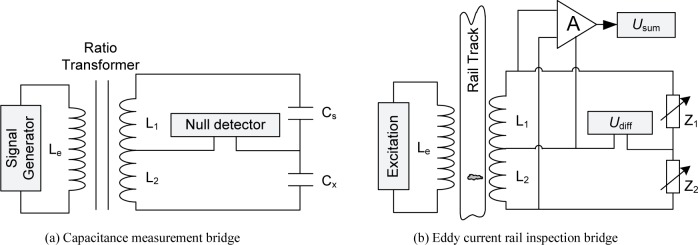
From impedance measurement bridge to rail inspection bridge.

AC bridge techniques are extensively used in precision impedance measurements [20]. For example, a precise and stable AC voltage ratio is realized using a three-winding transformer for comparing two capacitance standards, as shown in Fig. 1(a). The error signal of the four-arm bridge so formed can be measured using a lock-in amplifier and be further balanced out using voltage injection techniques, achieving parts in 10^9^ uncertainties. The key concept we borrow here for rail defect detection is the bridge balance. We use two detection coils L_1_ and L_2_ to form a four-arm bridge with two adjustable impedances Z_1_ and Z_2_, as shown in Fig. 1(b). The voltage ratio of the two detection coils then depends on the coupling between the excitation and detection coils and also on the rail to be inspected. First, the detection bridge is balanced for a defect-free rail by adjusting the impedance ratio, yielding a zero differential output voltage, *U*_diff_. It’s important to point out that due to the sensor symmetry, *U*_diff_ will remain nearly zero as long as the rail is defect-free, independent of the inevitable lift-off variations in field applications. As the sensor head passes through a defect region of the rail, defects will alter the voltage ratio of the detection coils and perturb the bridge balance, leading to a transient unbalance signal which can be monitored and analyzed continuously.

**Fig. 2:**
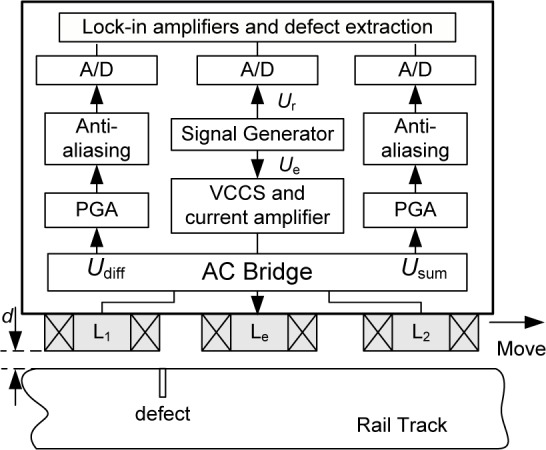
Structure of rail inspection bridge sensor and signal processing unit.

While the bridge balance is immune to lift-off variations when the rail is defect free, the differential signal *U*_diff_ associated with defects does depend on the lift-off distance, thus potentially affecting correct characterization of the defects. To compensate for the lift-off effect on the differential signal, we use the summing output, *U*_sum_, of the two detection coils, L_1_ and L_2_, to measure the lift-off distance, as shown in Fig. 1(b). The overall structure of the defect detection sensor with its signal processing circuits is depicted in Fig. 2. The excitation and detection coils are air-core, wrapped around plastic cylinders. The excitation coil is driven by a voltage-controlled current source (VCCS) combined with a signal generator.

The bridge output signals, *U*diff and *U*_sum_, are first conditioned by a programmable gain amplifier (PGA) and anti-aliasing filters. These signals, together with the reference signal, *U_r_*, are simultaneously digitized by A/D converters and are then demodulated.

**Fig. 3:**
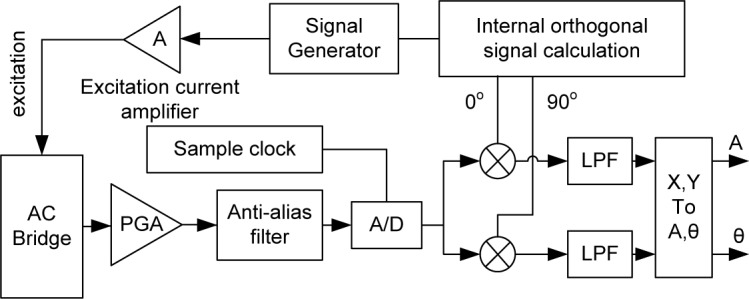
Diagram of digital lock-in amplifier algorithm.

The application environment of on-train rail inspection is characterized by various sources of electromagnetic noise. To reduce the noise, the excitation frequencies need to be carefully selected to avoid conflict with the frequencies and harmonics of the electric locomotives’ return current, track circuit working current, and railway wireless interphone broadcasting. And similar to AC bridge null detection, the digital lock-in algorithm is designed to demodulate the *U*_diff_ and *U*_sum_ signals in the rail inspection bridge. The digital lock-in amplifier algorithm is realized as shown in Fig. 3.

The internal orthogonal signal calculation, multiplier, and low pass filter (LPF) blocks are realized using C++ programming. The digital lock-in amplifier acts as a very narrow-band band pass filter (BPF) to reject interference noise to improve the rail defect detection ability.

## 4. Simulated Rail Inspection Experiment

### 4.1 Experimental Setup

**Fig. 4:**
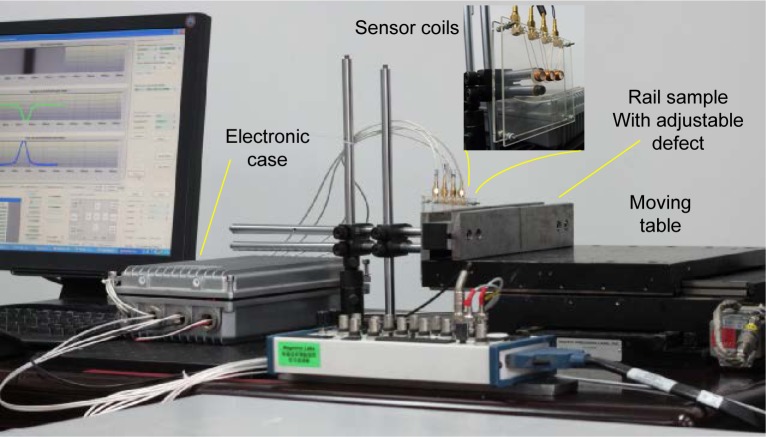
Experimental setup of rail inspection simulation.

An experimental setup, as shown in Fig. 4, has been developed to verify the new eddy current detection method. A rail sample with an adjustable crack is mounted on a computer controlled X-Y table. The X-axis movement of the table simulates the train’s movement along the track and the Y-axis movement simulates the vibrational bumping of the train. The excitation and detection coils are mounted on a frame that is fixed to an aluminum base board to keep the coil axes perpendicular to the rail surface. A custom electronic circuit is used to drive the excitation coil and amplify the detection coils’ signals. The conditioned signals are then fed to a NI-PXI data acquisition system^1^. In this paper, a standard transverse crack defect sample is used to test the efficacy of the inspection method. The transverse crack testing can be used for the four typical types of rail defects including TF (Transverse Fissure), CF (Compound Fissure), CH (Crushed Head), and DF (Detail Fracture) [21].

**Fig. 5:**
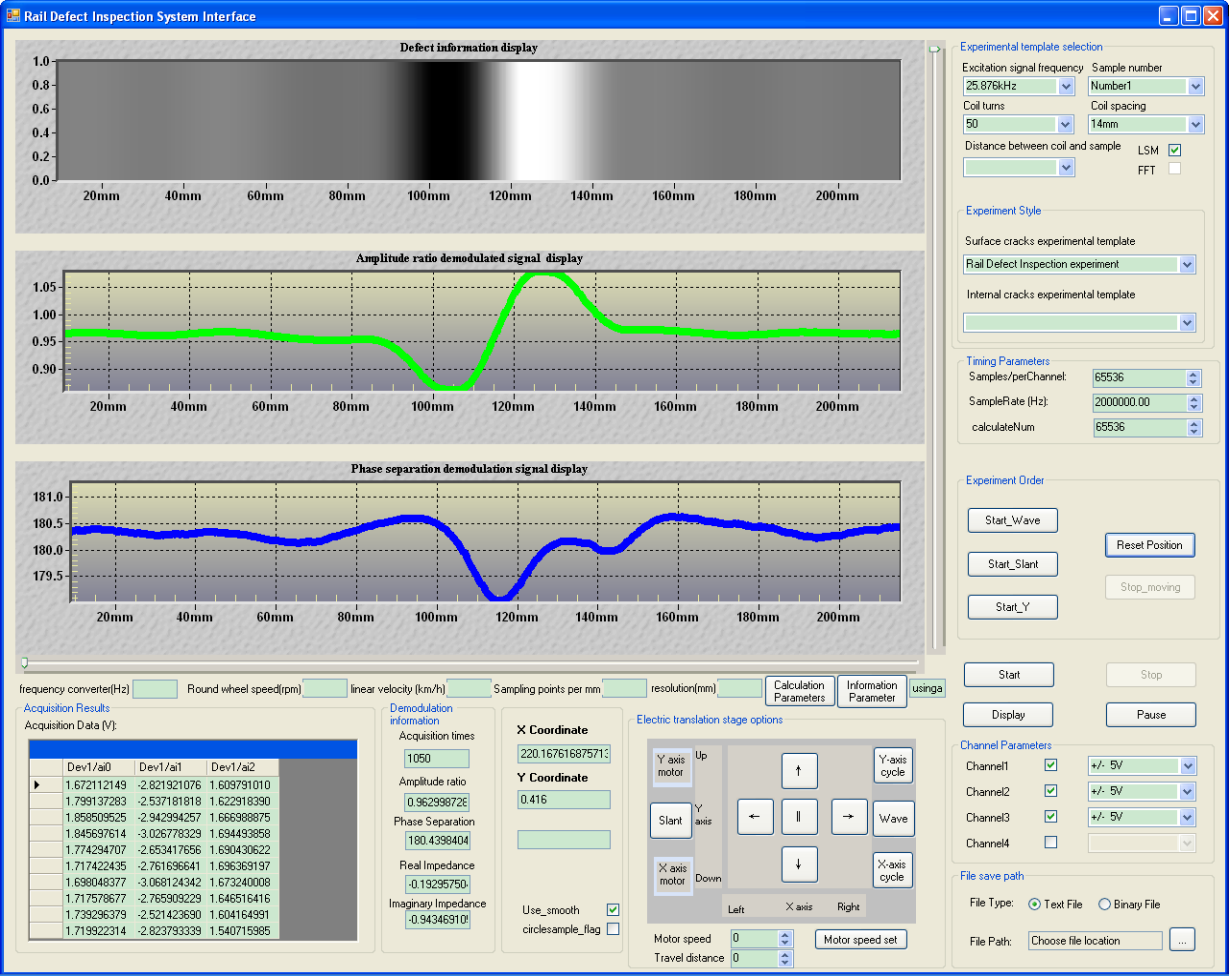
Interface of the rail inspection program.

The control program for the experimental setup is written in C++ with the user interface as shown in Fig. 5. The extracted defect information and demodulated signals are displayed as a function of the sensor’s moving distance. The excitation signal parameters and computer control functions for the X-Y table are also included in the main interface of the program.

**Fig. 6:**
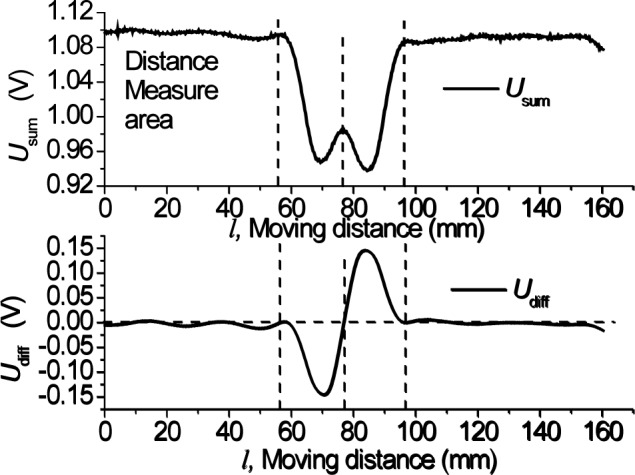
Bridge outputs when a cracked sample is scanned.

Using this experimental setup, a cross crack defect rail sample is tested by the sensor along a distance of 160 mm at a fixed lift-off distance. When the crack width is set to 1 mm, the bridge differential output *U*_diff_ and summary output *U*_sum_ are shown in Fig. 6. A transient *U*_diff_ waveform is clearly seen when the sensor passes over the defect while *U*_diff_ remains near zero when the sensor is over a defect-free region.

**Fig. 7:**
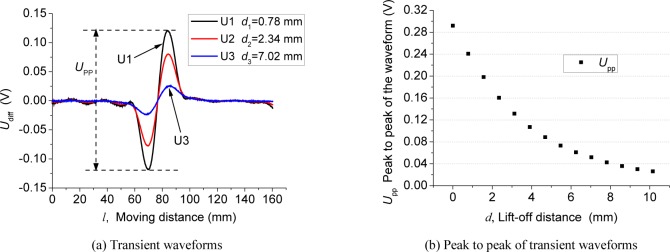
Defect transient waveforms with different lift-off distances.

The amplitude of the transient waveform which is used to characterize defects, however, strongly depends on the sensor’s lift-off distance. A series of experiments was performed with various lift-off distances with the same cracked rail sample. Shown in Fig. 7(a) are the transient waveforms corresponding to three different lift-off distances, *d*, which represents the gap between a sensor coil’s head and the rail sample surface. The shapes of these waveforms essentially remains the same, indicating that the lift-off affects only the amplitude. Shown in Fig. 7(b) is the measured peak-to-peak amplitude of the transient waveform, *U*_pp_, as a function of the lift-off distance.

**Fig. 8:**
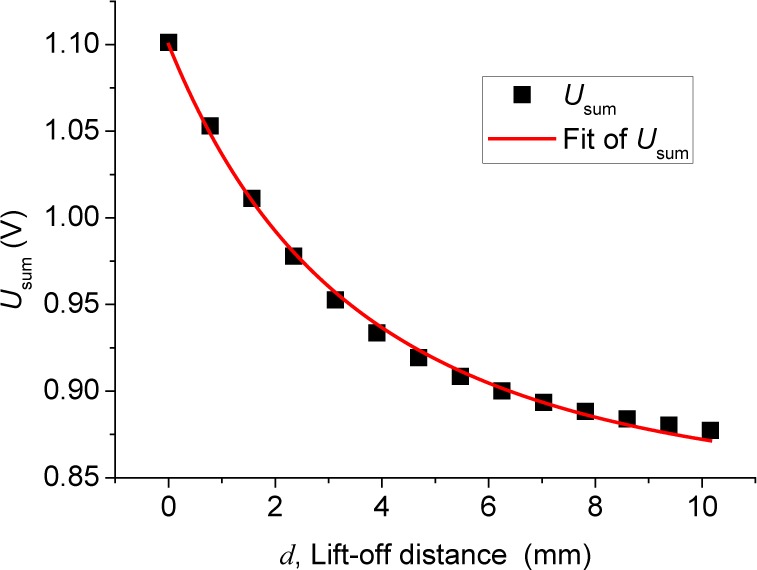
Relationship between *U*_sum_ and lift-off distance *d*.

In order to minimize the lift-off effect of the defect signals, we carefully studied the summing signal of the bridge output, *U*_sum_. Shown in Fig. 8 is the measured *U*_sum_ as a function of *d*, acquired simultaneously with the defect-induced data in Fig. 7(b). The relative uncertainty for the measured *U*_sum_ is about 0.02 %. The dominant component of the summing signal is a constant, *U*_sum_(∞), which results from direct coupling from the excitation coil and can be experimentally determined by moving the sensor far above the rail sample. The remainder of the summing signal, which decreases as the lift-off distance increases, is induced by the secondary eddy current. This dependence on the lift-off distance can be used to calibrate the differential signal.

**Fig. 9:**
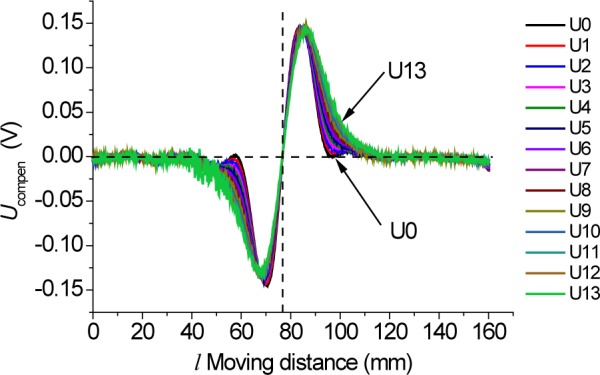
Compensated *U*_compen_ transient waveforms when passing the defective rail sample with different lift-off distances.

We use the following analytical formula to fit the data in Fig. 8:

(3) $$U_{\text{sum}}(d)=\frac{\alpha }{{\left(d+d_{0}\right)}^{2}}+\frac{\beta }{\left(d+d_{0}\right)}+U_{\text{sum}}(\infty )\equiv f(d)+U_{\text{sum}}(\infty ),$$

where *α* and *β* are the fitting parameters, and *d*_0_ is approximately the distance between the midpoint of the excitation coil and the midpoint of the eddy current distribution in the rail. Changing *d*_0_ slightly does not affect the overall fitting quality; it only affects the relative weight of the first and the second terms in Eq. (3). For the data shown in Fig. 8, *d*_0_ equals 8 mm and *U*_sum_(∞) is 0.85 V, and the fitting yields *α* = 23.1(V · mm^2^) and *β* = −0.88 (V · mm). *f* (*d*) so defined in Eq. (3) can be predetermined by measurements after the sensor geometry is fixed. This function reflects how the voltage component induced by the secondary eddy current depends on the lift-off distance and can be used to calibrate the differential signal as indicated in Eq. (4).

(4) $$U_{compen}(l)=U_{diff}(l)\times \frac{f(0)}{f(d)},$$

where *l* is the moving distance of the sensor along rail and *Ucompen*(*l*) is the compensated transient waveform data related to the defect. The lift-off distance is dynamically determined by constantly monitoring the summing signal. Experiments for a fixed 1 mm transverse crack rail sample have been done with different lift-off distances to verify the compensation method. Shown in Fig. 9 are 14 compensated defect transient waveforms with various lift-off distances ranging from 0.1 mm to 10.1 mm. The compensated defect-induced waveforms are essentially independent of the lift-off distance.

## 5. Conclusion

AC bridge techniques have been applied to the eddy current detection of rail defects. With an excitation coil and two detection coils configured as a three winding transformer, forming a classical four-arm bridge with two known impedances, the bridge differential error signal can sensitively detect rail defects using a digital lock-in amplifier algorithm. The lift-off effect of the bridge sensor due to vibrations can be measured using the summing signal of the detection coils and can be used to compensate the differential signal to more accurately reflect the defect characteristics. An experimental setup has been developed to test the design concept and verify the effectiveness of the bridge detection structure.

Future work includes using multiple frequencies to detect and characterize rail defects at various depths. A Field Programmable Gate Array (FPGA) could also be used to implement a faster digital lock-in amplifier algorithm to extract defect information and adapt to the speed of online on-train rail inspection. We are currently developing a new prototype for field applications. The prototype will be installed on an inspection vehicle with ultrasonic detection devices for side-by-side comparison of the two methods.

## Footnotes

1 Certain commercial equipment, instruments, or materials are identified in this paper to foster understanding. Such identification does not imply recommendation or endorsement by the National Institute of Standards and Technology, nor does it imply that the materials or equipment identified are necessarily the best available for the purpose.
